# A Synthetic Strategy for Conjugation of Paromomycin to Cell-Penetrating Tat(48-60) for Delivery and Visualization into Leishmania Parasites

**DOI:** 10.1155/2017/4213037

**Published:** 2017-02-14

**Authors:** Sira Defaus, Maria Gallo, María A. Abengózar, Luis Rivas, David Andreu

**Affiliations:** ^1^Department of Experimental and Health Sciences, Pompeu Fabra University, Barcelona Biomedical Research Park, 08003 Barcelona, Spain; ^2^Centro de Investigaciones Biológicas (CSIC), 28040 Madrid, Spain

## Abstract

A successful approach to deliver paromomycin, a poorly absorbed aminoglycoside antibiotic, to parasite cells is reported, based on selective protection of amino and hydroxyl groups followed by conjugation to a fluorolabeled, PEG-functionalized cell-penetrating Tat(48-60) peptide. The resulting construct is efficiently internalized into Leishmania cells, evidencing the fitness of cell-penetrating peptides as vectors for efficiently transporting low-bioavailability drugs into cells.

## 1. Introduction

**Scheme 1:**
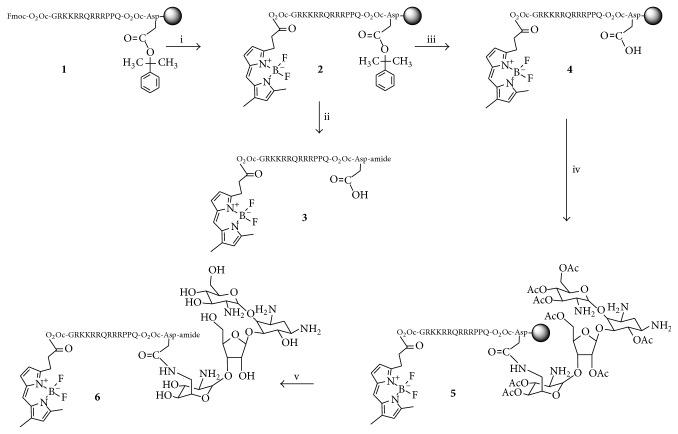
Synthetic approach to Tat-linked fluorescent PMM derivatives. Reagents and conditions: (i) piperidine/DMF (1 : 4), 1 + 20 min, then Bodi Fluor 488 acid (4 equiv), DIPCDI (4 equiv), CH_2_Cl_2_, 1 h + 1 h; (ii) TFA/H_2_O/TIS (95 : 2.5 : 2.5 v/v), 90 min, HPLC purification; (iii) TFA/CH_2_Cl_2_ (1%), 4 × 5 min, then DIEA/CH_2_Cl_2_ (5%), 4 × 5 min; (iv) 6,3′,4′,6′, 2′′,5′′,3′′′,4′′′-octa-O-acetyl-paromomycin (4 equiv), HBTU (4 equiv), DIEA (8 equiv), DMF, 2 h; (v) NaOMe/MeOH 0.5 M in CH_2_Cl_2_ (10 : 1), 2 h, then 15-crown-5/THF 0.05 M in acetic acid 20 : 1, 4 × 5 min, then TFA/H_2_O/TIS (95 : 2.5 : 2.5 v/v), 90 min, HPLC purification.

**Figure 1:**
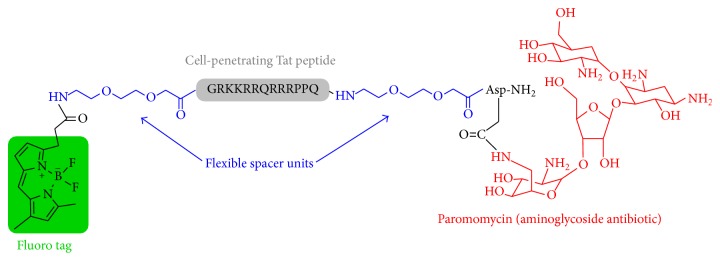
Structure of fluorolabeled PEG-modified PMM-Tat construct.

Aminoglycosides are broad spectrum antibiotics used as alternative antiparasitic agents that induce a deleterious effect on proliferation by interfering with the parasite's protein synthesis machinery [1, 2]. Specifically, paromomycin (PMM), a clinically approved aminoglycoside for the treatment of various bacterial and parasitic infections, is currently used alone [3] or in combination with other drugs [4, 5] to treat both cutaneous and visceral leishmaniasis, the latter a fatal disease. Recent reports on PMM selective antiparasitic effect on* Leishmania* ribosomes [6] and its low toxicity to mammalian cells have placed it on the list of essential medicines needed in a basic health system [7]. However, direct use of PMM in the clinic is hampered by its poor membrane permeability and consequently low intracellular accumulation. In order to overcome this limitation, PMM derivatives capable of crossing lipid bilayers without losing their activity are required. Cell-penetrating peptides (CPPs) are ideal candidates in this regard, by their ability to transport into cells a wide variety of cargo molecules, either covalently [8–11] or noncovalently bound [12–15]. For instance, miltefosine (hexadecylphosphocholine), a drug to which* Leishmania* is resistant due to poor accumulation, can be conjugated to a reference CPP such as Tat(48-60) to give a formulation with high absorption and parasiticidal activity that effectively defeats this resistance and expands the spectrum of susceptible trypanosomatids [16]. In an attempt to further expand this proof-of-principle, herein we report the synthesis of a PMM-CPP platform that also integrates a PEG-like spacer and a fluorescent tag for imaging purposes. Of various synthetic routes investigated, the only successful one (Scheme 1) relies on full on-resin assemblage of the target molecule, again highlighting the advantages of solid phase approaches for building complex biopolymer structures. Our results open the way to antiparasitic drugs with improved pharmacokinetic properties (Figure 1).

## 2. Materials and Methods

### 2.1. General

All reagents and solvents were used without further purification and handled according to manufacturer instructions. Fmoc-amino-3,6-dioxaoctanoic acid (O_2_Oc), other Fmoc-protected amino acids, and 2-(1H-benzotriazol-1-yl)-1,1,3,3-tetramethyluronium hexafluorophosphate (HBTU) were from Iris Biotech (Marktredwitz, Germany). Fmoc-Rink-amide ChemMatrix® resin was from* PCAS BioMatrix* (Montreal, Canada). HPLC-grade CH_3_CN and peptide synthesis-grade DMF, CH_2_Cl_2_,* N,N*-diethylisopropylamine (DIEA), and trifluoroacetic acid (TFA) were from Carlo Erba (Sabadell, Spain). Bodi Fluor™ 488 acid was from AAT Bioquest (Sunnyvale, CA). All other reagents were from Sigma-Aldrich (Madrid, Spain).

All procedures were carried out at room temperature unless otherwise indicated. Chromatographic purification of** 8**–**10** was done on glass columns packed with silica gel 60A (0.035–0.070 mm, Carlo Erba) eluted with the indicated solvents. TLC was carried out on Merck 5 × 20 cm silica gel 60 F254 plates (0.25 mm thick). Developed plates were visualized in a Spectroline ENF 260C/FE UV lamp (Spectronics Corp., Westbury, NY) and with a carbohydrate-specific reagent made of 1 g diphenylamine in 180 mL EtOH, 100 mL glacial HOAc, and 3 mL H_2_SO_4_. Organic solutions were dried over anhydrous MgSO_4_ and evaporated in R-200 rotavapor (Büchi). Melting points were determined in open glass capillaries in a Termovar F-05/76 apparatus (Reichert Technologies, Depew, NY) and are uncorrected. MALDI-TOF spectra were recorded in a Voyager DE-RP instrument (Perseptive Biosystems, Framingham, MA) using 2,4,6-trihydroxyacetophenone (THAP) as matrix. ^1^H-NMR spectra were obtained in a 500 MHz Inova spectrometer (Varian, Palo Alto, CA) in the indicated solvent. Chemical shifts are reported in *δ* (ppm) units relative to Me_4_Si as internal reference.

### 2.2. Peptide Synthesis

The target peptide was assembled at 50 *μ*moL scale on 0.106 g of Fmoc-Rink-amide ChemMatrix resin of 0.47 mmol/g substitution. The synthesis was performed in a Prelude instrument (Protein Technologies, Tucson, AZ) running optimized Fmoc protocols. Side-chain functions of Lys, Arg, Asp, and Asn were protected with Boc, 2,2,4,6,7-pentamethyldihydrobenzofuran-5-sulfonyl, 2-phenyisopropyl, and trityl groups, respectively. Double couplings were systematically performed with a 5-fold excess of Fmoc-amino acid or O_2_Oc in the presence of HBTU (5 eq) and DIEA (10 eq) with DMF as solvent for 2 × 30 min. Fmoc removal was done with 20% (v/v) piperidine in DMF for 2 × 2.5 min. Coupling and deprotection steps were separated by DMF washes (6 × 30 sec).

### 2.3. Peptide Analysis and Purification

Analytical reversed-phase HPLC was performed on Luna C_18_ columns (4.6 × 50 mm, 3 *μ*m, Phenomenex, Torrance, CA, USA) in LC-20AD system (Shimadzu, Kyoto, Japan). Solvents A and B were 0.045% and 0.036% (v/v) TFA in H_2_O and CH_3_CN, respectively. Elution was done with the indicated gradient of B into A over 15 min at 1 mL/min flow rate, with UV detection at 220 nm.

Preparative HPLC was performed on Luna C_18_ (10 × 250 mm, 10 *μ*m, Phenomenex) columns in a Shimadzu LC-8A instrument. Solvents A and B were 0.1% TFA in H_2_O and CH_3_CN, respectively, and elution was done with the indicated gradient of B into A over 30 min, at 7 mL/min flow rate, with UV detection at 220 nm.

Fractions of satisfactory purity (>95%) by analytical HPLC were pooled, lyophilized, and analyzed by HPLC-MS on C_18_ (4.6 × 150 mm column, 3.5 *μ*m, Phenomenex) in a Shimadzu LC-MS 2010EV instrument. Solvents A and B were 0.1% TFA (v/v) in H_2_O and 0.08% HCOOH in CH_3_CN, respectively. Elution was done with the indicated gradient of B into A over 15 min at 1 mL/min flow rate, with UV detection at 220 nm.

#### 2.3.1. Fmoc-O_2_ Oc-Tat(48-60)-O_2_Oc-Asp(O-2-PhiPr)-resin (**1**)

The Tat sequence (GRKKRRQRRRPPQ), elongated with a C-terminal Asp(O-2-PhiPr) residue and flanked by O_2_O spacer units at each end (Figure 1), was assembled in a Prelude synthesizer as described above. A mini cleavage with a small (~5 mg) amount of resin was performed to check the purity and identity of the resin-bound product.

*HPLC t*_*R*_ 8.24 min (5–60% linear gradient of B into A over 15 min).* ESI-MS m*/*z*: 782.85 [M+3H]^3+^, 587.40 [M+4H]^4+^, 470.15 [M+5H]^5+^ (calculated MW: 2343.69).

#### 2.3.2. Bodi Fluor-O_2_Oc-Tat(48-60)-O_2_Oc-Asp(O-2-PhiPr)-resin (**2**)

After incorporation of the N-terminal O_2_Oc residue, the Fmoc group was removed and Bodi Fluor 488 acid (58.4 mg, 200 *μ*moL, 4 eq over nominal substitution) was double-coupled with diisopropylcarbodiimide (DIPCDI, 200 *μ*moL, 4 eq) activation in CH_2_Cl_2_ for 1 h. A mini cleavage was performed to verify the purity and identity of the resin-bound product.

*HPLC t*_*R*_ 6.77 min (5–60% linear gradient of B into A over 15 min).* ESI-MS m*/*z*: 784.25 [M+3H]^3+^, 588.50 [M+4H]^4+^, 470.95 [M+5H]^5+^ (calculated MW: 2346.71).

#### 2.3.3. Bodi Fluor-O_2_Oc-Tat(48-60)-O_2_Oc-Asp(COOH)-amide (**3**)

Peptide-resin** 2** was treated with TFA/H_2_O/triisopropylsilane (95 : 2.5 : 2.5 v/v, 90 min) for full deprotection and cleavage. Crude peptide** 3** was isolated by precipitation with chilled diethyl ether, centrifuged at 4°C for 3 × 10 min, taken up in H_2_O, and lyophilized. It was purified by reverse-phase HPLC with a 5–60% linear gradient of B into A. Fractions of suitable purity (>95%) were pooled and tested for identity by MALDI-TOF MS.

*MALDI-TOF MS* (THAP, positive mode) *m*/*z*: 2419.96 [M+Na]^+^, 2434.96 [M+K]^+^ (calculated for C_100_H_169_BF_2_N_41_O_25_: 2396.50).

#### 2.3.4. Bodi Fluor-O_2_Oc-Tat(48-60)-O_2_Oc-Asp(COOH)-resin (**4**)

Peptide-resin** 2** was treated with 1% (v/v) TFA in CH_2_Cl_2_ (4 × 10 min) to orthogonally remove the O-2-PhiPr group, followed by neutralization with 5% DIEA in CH_2_Cl_2_ (4 × 5 min), to give** 4**. A mini cleavage would have been inconclusive and was thus omitted.

#### 2.3.5. Bodi Fluor-O_2_Oc-Tat(48-60)-O_2_Oc-Asp(6,3′,4′,6′,2′′,5′′,3′′′,4′′′-octa-O-acetyl-paromomycin)-resin (**5**)

Hydroxyl-protected PMM** 10** (190.4 mg, 200 *μ*moL) was coupled to the Asp *β*-carboxyl group of** 4** in the presence of HBTU (75.8 mg, 200 *μ*moL) and DIEA (70 *μ*L, 400 *μ*moL) in DMF for 2 h, followed by extensive DMF and CH_2_Cl_2_ washes. A mini cleavage was performed to verify the purity and identity of the resin-bound product.

*HPLC t*_*R*_ 7.17 min (5–60% linear gradient of B into A over 15 min).* ESI-MS m*/*z*: 821.85 [M+4H]^4+^, 657.75 [M+5H]^+5^, 548.30 [M+6H]^+6^, 470.05 [M+7H]^+7^ (MW calculated: 3278.65).

#### 2.3.6. Bodi Fluor-O_2_Oc-Tat(48-60)-O_2_Oc-Asp(paromomycin)-amide (**6**)

**Figure 2:**
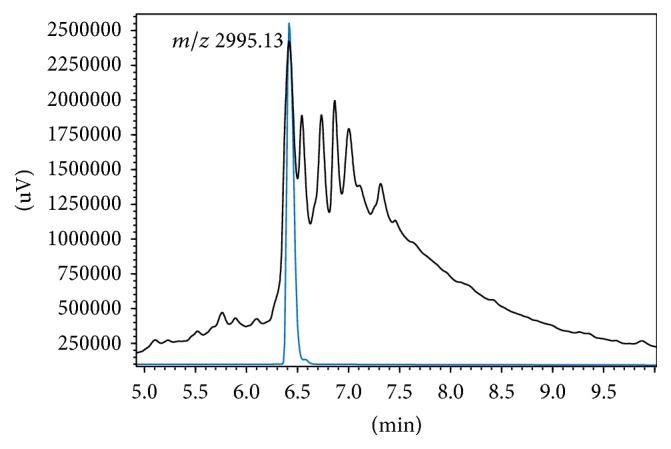
HPLC analysis of fluorolabeled PEG-modified PMM-Tat conjugate** 6**. Black trace: crude product after TFA acidolysis. Blue trace: HPLC-purified product.

Resin** 5** was suspended in 20 mL CH_2_Cl_2_ and treated with 2 mL of 0.5 M sodium methoxide in MeOH for 2 h, followed by washes with a 0.05 M solution of 15-crown-5 in THF containing 5% (v/v) HOAc to remove sodium ions. The resin was then treated with TFA/H_2_O/triisopropylsilane (95 : 2.5 : 2.5 v/v, 90 min) for full deprotection and cleavage. The target product was isolated by precipitation with chilled diethyl ether, centrifuged for 3 × 10 min at 4°C, taken up in H_2_O, and lyophilized. Analytical HPLC revealed a complex mixture (black trace, Figure 2) in which only a product with the expected mass of** 6** was observed and could be successfully purified by preparative HPLC (blue trace).

*HPLC t*_*R*_ 6.4 min (5–60% linear gradient of B into A over 15 min).* ESI-MS m*/*z*: 983.40 [M+3H]^+3^, 737.75 [M+4H]^+4^, 590.40 [M+5H]^+5^, 492.20 [M+6H]^+6^, 422.00 [M+7H]^+7^, 369.35 [M+8H]^+8^ (MW calculated: 2944.33).* MALDI-TOF MS* (THAP, positive mode) *m*/*z*: 3017.6 [M+Na]^+^, 3033.6 [M+K]^+^ (calculated for C_123_H_213_BF_2_N_46_O_38_: 2995.13).

#### 2.3.7. 1,3,2′,2′′′,6′′′-Pentakis(N-tert-butyloxycarbonyl)-paromomycin (**8**)

700 mg PMM sulfate** 7** (0.98 mmoL, 1 eq) was dissolved in 9.8 mL H_2_O, 4.9 mL MeOH, and 1.6 mL TEA (11.8 mmoL, 12 eq). Then 3.4 g of Boc_2_O (14.7 mmoL, 15 eq) dissolved in 4.9 mL MeOH was added dropwise to the stirred solution and the mixture was heated at 60°C for 24 h. After cooling, TEA and MeOH were removed by evaporation, the aqueous residue was extracted with EtOAc (3 × 10 mL), and the organic layers were washed with brine (3 × 5 mL), dried, and evaporated to a white solid that was purified on silica gel (CH_2_Cl_2_ to 80 : 20 CH_2_Cl_2_/MeOH) giving** 8** (594 mg, 83% yield).

*TLC* (CH_2_Cl_2_/CH_3_OH, 9 : 1) Rf 0.5. ^*1*^*H-NMR* (DMSO-*d*_6_, 500 MHz, ppm) only most relevant signals: *δ* = 6.78 (d, *J* = 5.0 Hz, 1 H), 6.74 (t, *J* = 10.0 Hz, 1H), 6.62 (s, 1 H), 6.20 (d, *J* = 10.0 Hz, 1 H), 5.81 (d, *J* = 10.0 Hz, 1 H) (5 × NHBoc), 1.35 (s, 45 H, CH_3_* t*Bu).* MALDI-TOF MS* (THAP, positive mode) *m*/*z*: 1138.2 [M+Na]^+^, 1154.2 [M+K]^+^ (calculated for C_48_H_85_N_5_O_24_: 1115.56).

#### 2.3.8. 6,3′,4′,6′,2′′,5′′,3′′′,4′′′-Octa-O-acetyl-1,3,2′,2′′′,6′′′-pentakis(N-tertbutyloxycarbo-nyl)-paromomycin (**9**)

780 *μ*L of Ac_2_O (8.25 mmoL, 2 × 8 OH = 16 eq) was added to a stirred solution containing 575 mg of** 8** (0.52 mmoL, 1 eq) in 2.9 mL of anhydrous pyridine in the presence of a catalytic amount of* N,N*-dimethylaminopyridine. After 20 h stirring at room temperature, the reaction mixture was diluted with 20 mL CH_2_Cl_2_ and the organic layer was washed with 10% (w/v) citric acid (1 × 20 mL), saturated NaHCO_3_ (3 × 15 mL), and brine (3 × 15 mL), dried, and evaporated to a white residue that was purified by column chromatography (hexane/ethyl acetate 1 : 1) to give** 9** (380 mg, 65%).

*TLC* (hexane/ethyl acetate 1 : 1) *R*_*f*_ 0.45. ^*1*^*H-NMR* (DMSO-*d*_6_, 500 MHz, ppm) only most relevant signals: *δ* = 6.87 (d, *J* = 10.0 Hz, 1 H), 6.80 (d, *J* = 10.0 Hz, 1 H), 6.06 (d, *J* = 10.0 Hz, 1 H), 5.57 (s, 1 H), 5.38 (d, *J* = 10.0 Hz, 1 H) (5 × NHBoc), 2.07, 2.04, 1.98, 1.92, 1.86 (5 s, 24 H, CH_3_O-), 1.35 (s, 45 H, CH_3_* t*Bu).* MALDI-TOF MS* (THAP, positive mode) *m*/*z*: 1474.6 [M+Na]^+^, 1490.6 [M+K]^+^ (calculated for C_64_H_101_N_5_O_32_: 1452.51).

#### 2.3.9. 6,3′,4′,6′,2′′,5′′,3′′′,4′′′-Octa-O-acetyl-paromomycin (**10**)

360 mg of** 9** (0.248 mmol, 1 eq) was treated with 36 mL of 40% TFA in CH_2_Cl_2_ for 2 h at room temperature. CH_2_Cl_2_ was evaporated and a solid was precipitated by the addition of chilled diethyl ether, centrifuged, taken up in H_2_O, and lyophilized to give 236 mg of** 10** (100% yield, 25% from PMM). After satisfactory characterization by analytical HPLC and MS, it was used without further purification.

*HPLC t*_*R*_ 7.68 min (0–50% linear gradient of B into A over 15 min, 50°C). ^*1*^*H-NMR* (DMSO-*d*_6_, 500 MHz, ppm) only most relevant signals: *δ* = 2.09, 2.08, 2.07, 1.98, 1.83 (5 s, 24H, CH_3_O-), 8.46 (5 × NH_2_, broad band).* ESI-MS m*/*z*: 952.35 [M+H]^+1^, 477.10 [M+2H]^+2^ (MW calculated: 951.92).* MALDI-TOF MS* (THAP, positive mode) *m*/*z*: 974.5 [M+Na]^+^, 990.5 [M+K]^+^ (calculated for C_39_H_61_N_5_O_22_: 951.92).

### 2.4. Parasite Culture and Confocal Microscopy

*Leishmania donovani* promastigotes (strain MHOM/SD/00/1S-2D) were resuspended in Hanks' medium supplemented with 10 mM D-glucose (HBSS-Glc) at 2 × 10^7^ cells/mL. Afterwards, 9 *μ*M Bodi Fluor 488-labeled PMM-Tat conjugate** 6** was added to the parasite suspension and incubated for 4 h in HBSS-Glc. After incubation, non-incorporated** 6** was removed by washing the cells with HBSS-Glc. Finally, cells were labeled with DAPI (5 *μ*g/mL, 20 min) before their observation, unfixed, in a Leica TCS–SP2 ABOS confocal laser scanning microscope.

## 3. Results and Discussion

Although conjugation of organic (drug) molecules to peptides is ideally done while the peptide is bound to the solid support [17], the many hydroxyl and amino groups of aminoglycosides render them poorly soluble in the organic solvents where solid phase reactions are best run. In view of this limitation, exploratory attempts to link water-soluble PMM to Tat peptide, in aqueous medium and at a pH favoring conjugation through a peptide bond, were initially done. Specifically, a CPP with the Tat(48-60) sequence plus an extra C-terminal Asp residue (**1**, Scheme 1) was assembled on a Rink-amide resin by Fmoc-based solid phase synthesis methods [18]. One residue each of amino-3,6-dioxaoctanoic acid (O_2_Oc), a nontoxic, nonimmunogenic flexible spacer, was introduced to separate the canonic CPP sequence from Asp and the N-terminal tag (Figure 1). For Asp *β*-carboxyl protection, two orthogonally removable groups, N-[1-(4,4-dimethyl-2,6-dioxocyclohexylidene)-3-methylbutyl]amino benzyl (Dmab) and 2-phenylisopropyl (2-PhiPr), were evaluated. The Dmab group did not live up to its promise: selective deprotection with 2% hydrazine/DMF gave rise to a complex array of products, none of them assignable to a structure readily inferable from the hitherto assembled sequence; hence the approach was discarded. In contrast, 2-PhiPr was cleanly removed by 1% TFA/CH_2_Cl_2_ in the presence of all other tBu-based protecting groups. In addition and in order to monitor PMM distribution and localization within the parasites, a fluorescent tag was required at the N-terminus. To this end, a Bodipy [19] analogue with ideal hydrophobic properties for staining membranes and other lipid-based structures was chosen. Thus, Bodi Fluor 488 acid was coupled to the O_2_Oc-elongated Tat peptide resin (**1**, Scheme 1) to give intermediate** 2**, which could either be converted to free Bodi Fluor-Tat(48-60)-Asp peptide** 3** by acidolysis and HPLC purification (step ii, Scheme 1) or further elaborated for on-resin conjugation (see Scheme 1).

In the attempt to conjugate** 3** and PMM in aqueous solution, 1 eq each of peptide and 1-ethyl-3-(3-dimethylaminopropyl)carbodiimide, a water-soluble coupling agent, were dissolved in phosphate buffer, pH 5; PMM was added and the pH raised to 8 to favor amide formation between the single Asp side-chain carboxyl and the least hindered primary amine of the aminoglycoside. HPLC-MS analysis after 90 min reaction and acidification at pH 2 revealed a complex crude in which the species with the expected mass was present but in such low amounts and purity to render workup unfeasible. The solution approach was therefore ruled out in favor of amide bond formation between the Asp side-chain carboxyl of resin-bound** 4** and a suitably protected version of PMM with a single free amino group.

**Scheme 2:**
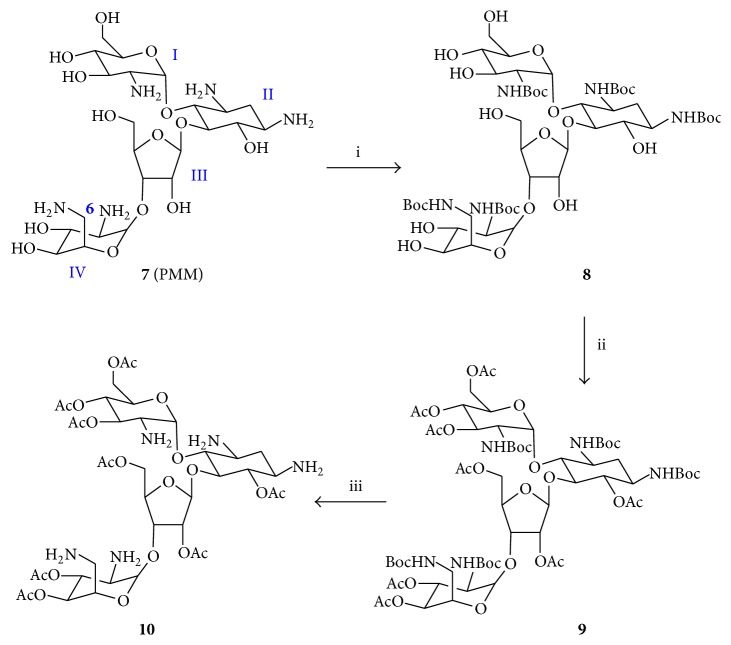
Preparation of hydroxyl-protected paromomycin. Reagents and conditions: (i) Boc_2_O (15 equiv), Et_3_N (12 equiv), MeOH, H_2_O, 24 h, 60°C, 64%; (ii) Ac_2_O (16 equiv), anh. pyridine, DMAP, 20 h, R.T., 78%; (iii) TFA/CH_2_Cl_2_ (40%), 2 h, R.T., 100%.

Initial attempts to protect the eight hydroxyl groups of PMM in the presence of amino groups by means of silylating reagents (e.g., trimethylsilyl chloride or iodide) allegedly selective for alcohols were unsuccessful. LC-MS analysis invariably showed heterogeneous mixtures of O- and N-protected PMM derivatives unsuitable for subsequent conjugation to** 4**. In view of these difficulties, a controlled, multistep procedure leading to selectively O-protected PMM was undertaken (Scheme 2) [20].

Treatment of PMM monosulfate (**7**) with Boc_2_O and TEA yielded (64%) the pentacarbamate** 8**, which was acetylated to give fully protected** 9** (78%). Then, Boc removal with 40% TFA/CH_2_Cl_2_ led to selectively O-protected octa-O-acetyl-PMM (**10**). Next, amide bond formation between (preferentially) the most accessible amino group of** 10** (i.e., N6′′′ in ring IV, Scheme 2) and the activated Asp *β*-carboxyl of** 4** was undertaken. The resulting resin-bound PMM-peptide conjugate** 5** was treated with 0.5 M sodium methoxide in MeOH and repeatedly washed with a sodium-complexing solution to remove O-acetyl groups [20].

Finally, the target compound was obtained upon acidolytic removal of peptide side-chain protecting groups and cleavage from the resin. The resulting crude (Figure 2) was complex, as expected from an elaborate stepwise process, but HPLC purification and lyophilization allowed 15.5 mg (10% yield) of an HPLC homogeneous product with a mass of 2,995.13 Da to be isolated. None of the accompanying peaks in Figure 2 had this same mass; hence they were attributed to by-products or intermediates built up over the solid phase steps. While the possibility of regioisomers coeluting with** 6** in the peak at 6.4 min must not be ruled out, the structure of** 6** as the purified product can be rather safely inferred as the result of preferential attack at the sterically less hindered amino group of** 10**.

**Figure 3:**
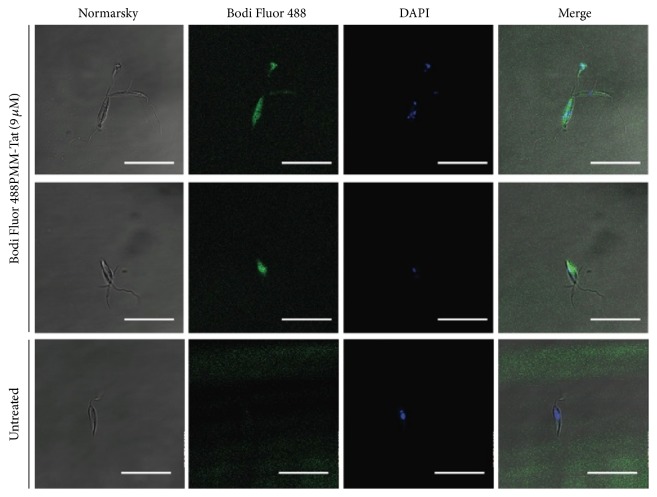
Fluorescence of* Leishmania donovani* promastigotes incubated with Bodi Fluor 488-labeled PMM-Tat conjugate (9 *μ*M, 4 h, 26°C) and stained with DAPI (5 *μ*g/mL) immediately before observation, unfixed. Settings: conjugate (green, *λ*_ex_ = 488 nm/*λ*_em_ = 520 nm); DAPI (blue, *λ*_ex_ = 350 nm/*λ*_em_ = 460 nm). Bar = 20 *μ*m. Experiment is representative of two other ones performed independently.

The intracellular uptake of the Bodi Fluor 488-labeled PMM-Tat conjugate** 6** by* L. donovani promastigotes* was investigated by confocal fluorescence microscopy (Figure 3). The internalization and cellular pattern showed the conjugate can enter the parasite, with a diffuse pattern in all the cytoplasm.

## 4. Conclusions

In summary, we have successfully synthesized a CPP-linked fluorescent PMM derivative by a synthetic approach that combines selective protection of the aminoglycoside and solid phase peptide synthesis. This presentation allows fast, effective PMM delivery into* Leishmania* cells, thus validating our approach as a suitable strategy to overcome poor drug accumulation, a key factor for PMM resistance in* Leishmania* [21]. Additional prospective advantages are the possibility to use CPP conjugates to target specific subcellular compartments by addition of import sequences modifying physicochemical properties [22]. Also, CPP-mediated delivery of PMM may increase biodistribution into deeper layers of the* Leishmania* ulcer, the CPP acting as a skin vehicle for the drug [23, 24].

## Supplementary Material

Supporting Information, available on line, includes experimental procedures for compounds 1-10 and their ^1^H-NMR, HPLC and mass spectral data, as well as protocols for parasite culture.

## References

[1] François B., Szychowski J., Adhikari S. S. (2004). Antibacterial aminoglycosides with a modified mode of binding to the ribosomal-RNA decoding site. *Angewandte Chemie—International Edition*.

[2] Shalev M., Kondo J., Kopelyanskiy D., Jaffe C. L., Adir N., Baasov T. (2013). Identification of the molecular attributes required for aminoglycoside activity against *Leishmania*. *Proceedings of the National Academy of Sciences of the United States of America*.

[3] Jamil K. M., Haque R., Rahman R. (2015). Effectiveness study of paromomycin IM injection (PMIM) for the treatment of visceral leishmaniasis (VL) in Bangladesh. *PLoS Neglected Tropical Diseases*.

[4] Das S., Rani M., Rabidas V., Pandey K., Sahoo G. C., Das P. (2014). TLR9 and MyD88 are crucial for the maturation and activation of dendritic cells by paromomycin-miltefosine combination therapy in visceral leishmaniasis. *British Journal of Pharmacology*.

[5] Ben Salah A., Ben Messaoud N., Guedri E. (2013). Topical paromomycin with or without gentamicin for cutaneous leishmaniasis. *The New England Journal of Medicine*.

[6] Fernández M. M., Malchiodi E. L., Algranati I. D. (2011). Differential effects of paromomycin on ribosomes of Leishmania Mexicana and mammalian cells. *Antimicrobial Agents and Chemotherapy*.

[7] WHO (2015). *19th WHO Model List of Essential Medicines*.

[8] Tyagi M., Rusnati M., Presta M., Giacca M. (2001). Internalization of HIV-1 tat requires cell surface heparan sulfate proteoglycans. *Journal of Biological Chemistry*.

[9] Beaudette T. T., Cohen J. A., Bachelder E. M. (2009). Chemoselective ligation in the functionalization of polysaccharide-based particles. *Journal of the American Chemical Society*.

[10] Dutot L., Lécorché P., Burlina F. (2010). Glycosylated cell-penetrating peptides and their conjugates to a proapoptotic peptide: preparation by click chemistry and cell viability studies. *Journal of Chemical Biology*.

[11] Steven V., Graham D. (2008). Oligonucleotide conjugation to a cell-penetrating (TAT) peptide by Diels-Alder cycloaddition. *Organic and Biomolecular Chemistry*.

[12] Crombez L., Morris M. C., Heitz F., Divita G. (2011). A non-covalent peptide-based strategy for ex vivo and in vivo oligonucleotide delivery. *Methods in Molecular Biology*.

[13] Deshayes S., Konate K., Aldrian G., Crombez L., Heitz F., Divita G. (2010). Structural polymorphism of non-covalent peptide-based delivery systems: highway to cellular uptake. *Biochimica et Biophysica Acta*.

[14] Henriques S. T., Costa J., Castanho M. A. R. B. (2005). Translocation of *β*-galactosidase mediated by the cell-penetrating peptide pep-1 into lipid vesicles and human HeLa cells is driven by membrane electrostatic potential. *Biochemistry*.

[15] Lindberg S., Muñoz-Alarcón A., Helmfors H. (2013). PepFect15, a novel endosomolytic cell-penetrating peptide for oligonucleotide delivery via scavenger receptors. *International Journal of Pharmaceutics*.

[16] De La Torre B. G., Hornillos V., Luque-Ortega J. R. (2014). A BODIPY-embedding miltefosine analog linked to cell-penetrating Tat(48-60) peptide favors intracellular delivery and visualization of the antiparasitic drug. *Amino Acids*.

[17] Mäde V., Els-Heindl S., Beck-Sickinger A. G. (2014). Automated solid-phase peptide synthesis to obtain therapeutic peptides. *Beilstein Journal of Organic Chemistry*.

[18] Fields G. B., Noble R. L. (1990). Solid phase peptide synthesis utilizing 9-fluorenylmethoxycarbonyl amino acids. *International Journal of Peptide and Protein Research*.

[19] Ulrich G., Ziessel R., Harriman A. (2008). The chemistry of fluorescent bodipy dyes: versatility unsurpassed. *Angewandte Chemie—International Edition*.

[20] Rademann J., Schmidt R. R. (1997). Repetitive solid phase glycosylation on an alkyl thiol polymer leading to sugar oligomers containing 1,2-trans- and 1,2-cis-glycosidic linkages. *Journal of Organic Chemistry*.

[21] Bhandari V., Sundar S., Dujardin J. C., Salotra P. (2014). Elucidation of cellular mechanisms involved in experimental paromomycin resistance in *Leishmania donovani*. *Antimicrobial Agents and Chemotherapy*.

[22] Yousif L. F., Stewart K. M., Kelley S. O. (2009). Targeting mitochondria with organelle-specific compounds: strategies and applications. *ChemBioChem*.

[23] Chen Y., Wang M., Fang L. (2013). Biomaterials as novel penetration enhancers for transdermal and dermal drug delivery systems. *Drug Delivery*.

[24] Lopes L. B., Carvalho V. F. M., de Lemos D. P. (2015). Potential of peptide-based enhancers for transdermal delivery. *Current Pharmaceutical Design*.

