# Supplementary material for: A Synthetic Strategy for Conjugation of Paromomycin to Cell-Penetrating Tat(48-60) for Delivery and Visualization into Leishmania Parasites
Source: Int J Pept. 2017 Feb 14;2017:4213037. doi: 10.1155/2017/4213037 (PMC5329690; doi:10.1155/2017/4213037)
Supplement: Supplementary file 1 — Supporting Information, available on line, includes experimental procedures for compounds 1-10 and their 1H-NMR, HPLC and mass spectral data, as well as protocols for parasite culture. [file 4213037.f1.pdf]

Supporting Information

A synthetic strategy for conjugation of paromomycin to cell-penetrating Tat(48-60) for delivery and visualization into *Leishmania* parasites

Sira Defaus[1†], Maria Gallo[1†], María A. Abengózar[2], Luis Rivas[2], David Andreu[1]*

Contents

1. **Materials & Methods**
   - 1.1. General
   - 1.2. Peptide synthesis
   - 1.3. Peptide analysis and purification

2. **Experimental data**
   - 2.1. Fmoc-O_2Oc-Tat(48-60)-O_2Oc-Asp(O-2-PhiPr)- resin (**1**)
   - 2.2. BodiFluor-O_2Oc-Tat(48-60)-O_2Oc-Asp(O-2-PhiPr)-resin (**2**)
   - 2.3. BodiFluor-O_2Oc-Tat(48-60)-O_2Oc-Asp(COOH)-amide (**3**)
   - 2.4. BodiFluor-O_2Oc-Tat(48-60)-O_2Oc-Asp(COOH)-resin (**4**)
   - 2.5. BodiFluor-O_2Oc-Tat(48-60)-O_2Oc-Asp(6,3',4',6',2'',5'',3''',4'''-octa-O-acetyl-paromomycin)-resin (**5**)
   - 2.6. BodiFluor-O_2Oc-Tat(48-60)-O_2Oc-Asp(paromomycin)-amide (**6**)
   - 2.7. 1,3,2',2''',6'''-pentakis(N-*tert*-butoxycarbonyl)-paromomycin (**8**)
   - 2.8. 6,3',4',6',2'',5'',3''',4'''-octa-O-acetyl-1,3,2',2''',6'''-pentakis(N-*tert*-butyloxycarbonyl)-paromomycin (**9**)
   - 2.9. 6,3',4',6',2'',5'',3''',4'''-octa-O-acetyl-paromomycin (**10**)

3. **Spectral data**
   - 3.1. ^{1}H NMR spectrum of aminoglycosides derivatives (**8**-**10**)
   - 3.2. HPLC and MS of **10** and final product **6**

4. **Parasite culture and confocal microscopy**

1. Materials & Methods

1.1. General

All reagents and solvents were commercially available, used without further purification and handled according to material safety data sheet of the supplier. Fmoc-amino-3,6-dioxaoctanoic acid (O_2Oc), other Fmoc-protected amino acids and HBTU were from Iris Biotech (Marktredwitz Germany). Fmoc-Rink amide ChemMatrix® resin was from PCAS BioMatrix (Montreal, Canada). HPLC-grade CH_3CN and peptide synthesis-grade DMF, CH_2Cl_2, DIEA and TFA were from Carlo Erba (Sabadell, Spain). All other reagents were from Sigma-Aldrich (Madrid, Spain).

All procedures were carried out at room temperature unless otherwise indicated. Chromatographic purification of **8**-**10** was performed on glass columns packed with silica gel 60A (0.035-0.070 mm, Carlo Erba, Sabadell, Spain) using the reported eluents. TLC was carried out on Merck 5 × 20 cm silica gel 60 F254 plates (0.25 mm thick). Developed plates were visualized in a Spectroline ENF 260C/FE UV lamp and with a carbohydrate-specific reagent made of 1 g of diphenylamine in 180 mL EtOH, 100 $MgSO_4$ and rotary-evaporated in a R-200 rotavapor (Büchi). Melting points were determined in open glass capillaries in a Termovar F-05/76 (Reichert) apparatus and are uncorrected. MALDI-TOF spectra were recorded in a Voyager DE-RP instrument (Perseptive Biosystems) using 2,4,6-trihydroxyacetophenone as matrix. ^1H-NMR spectra were obtained at 500 MHz in a Varian Inova spectrometer in the indicated solvent. Chemical shifts are reported in δ (ppm) units relative to Me_4Si as internal reference.

1.2. Peptide synthesis

The target peptide was assembled at 50 μmol scale on 0.106 g of Fmoc-Rink-amide ChemMatrix resin of 0.47 mmol/g substitution. The synthesis was performed in a Prelude instrument (Protein Technologies, Tucson, AZ) running optimized Fmoc protocols. Side-chain functions were protected with Boc (Lys), 2,2,4,6,7 pentamethyldihydrobenzofuran-5-sulfonyl (Arg), 2-phenylisopropyl (Asp) and trityl (Asn) groups. Double couplings were systematically performed with a 5-fold excess of Fmoc-amino acid or O_2Oc in the presence of 2-(1H-benzotriazol-1-yl)-1,1,3,3 tetramethyluronium hexafluorophosphate (HBTU, 5-fold excess) and DIEA (10-fold excess) with DMF as solvent for 5 + 5 min. Fmoc group removal was done by two sequential treatments with 20% (v/v) piperidine in DMF for 2.5 min. Coupling and deprotection steps were separated by DMF washes (6 × 30 min). All peptide synthesis procedures were run at room temperature.

1.3. Peptide analysis and purification

Analytical reversed-phase HPLC was performed on Luna C_{18} columns (4.6 × 50 mm, 3 µm, Phenomenex, Torrance, CA, USA) in a LC-20AD system (Shimadzu, Kyoto, Japan). Solvents A and B were 0.045% and 0.036% (v/v) TFA in H_2O and CH_3CN, respectively. Elution was done with the indicated gradient of solvent B into A over 15 min at 1 mL/min flow rate, with UV detection at 220 nm.

Preparative HPLC was performed on Luna C_{18} (10 x 250 mm, 10 µm, Phenomenex) columns in a Shimadzu LC-8A instrument. Solvents A and B were 0.1 % TFA in H_2O and CH_3CN, respectively, and elution was done with indicated gradient of solvent B into A over 30 min, at 7 mL/min flow rate with UV detection at 220 nm.

Fractions of satisfactory purity (>95%) by analytical HPLC were pooled, lyophilized and analyzed for identity by HPLC-MS on C_{18} (4.6 x 150 mm column, 3.5 µm, Phenomenex) in a Shimadzu LC-MS 2010EV instrument. Solvent A was 0.1% TFA (v/v) in H_2O, solvent B was 0.08% HCOOH in CH_3CN. Elution was done with indicated gradient of solvent B into A over 15 min at 1 mL/min flow rate, with UV detection at 220 nm.

2. Experimental data

2.1. Fmoc-O_2Oc-Tat(48-60)-O_2Oc-Asp(O-2-PhiPr)-resin (**1**)

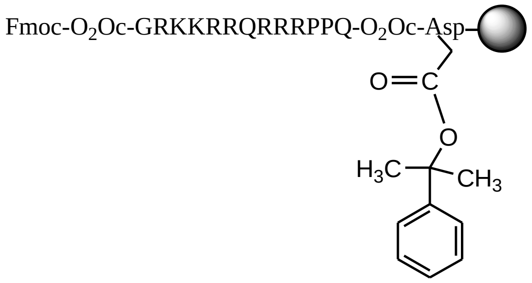

The Tat sequence, flanked by an O_2O spacer unit at each end and with a C-terminal Asp(O-2-PhiPr) residue was assembled in a Prelude synthesizer as described in 1.2. above. A mini cleavage with a small (~5 mg amount of resin) was performed to check the purity and identity of the resin-bound product.

HPLC t_R 8.24 min (5-60% linear gradient of B into A over 15 min). **ESI-MS** m/z: 782.85 $[M+3H]^{3+}$, 587.40 $[M+4H]^{4+}$, 470.15 $[M+5H]^{5+}$ (MW calculated: 2343.69).

2.2 BodiFluor-O_2Oc-Tat(48-60)-O_2Oc-Asp(O-2-PhiPr)-resin (**2**)

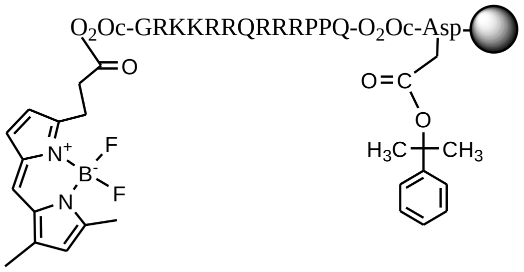

After incorporation of the N-terminal O_2Oc residue, the Fmoc group was removed with piperidine (see 1.2.) and BodiFluor™ 488 acid (58.4 mg, 200 µmol, 4-fold excess over nominal substitution) was double-coupled with DIPCDI (200 µmol, 4-fold excess) activation in CH_2Cl_2 for 1h + 1h. A mini cleavage was performed to verify the purity and identity of the resin-bound product.

HPLC t_R 6.77 min (5-60% linear gradient of B into A over 15 min). **ESI-MS** m/z: 784.25 $[M+3H]^{3+}$, 588.50 $[M+4H]^{4+}$, 470.95 $[M+5H]^{5+}$ (MW calculated: 2346.71).

2.3. BodiFluor-O_2Oc-Tat(48-60)-O_2Oc-Asp(COOH)-amide (**3**)

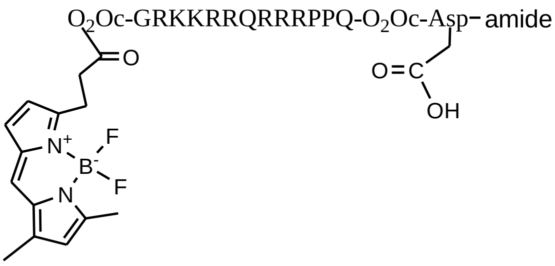

Peptide resin **2** was treated with TFA/H_2O/TIS (95:2.5:2.5 v/v, 90 min) for full deprotection and cleavage. Crude peptide **3** was isolated from the cleavage solution by precipitation with chilled diethyl ether, centrifuged at 4ºC for 3 × 10 min, taken up in H_2O and lyophilized. It was purified by reverse-phase HPLC with a 5-60% linear gradient of solvent B into A as described in 1.3. Fractions of suitable purity (>95 %) were pooled and tested for identity by MALDI-TOF MS.

MALDI-TOF MS (THAP, positive mode) m/z: 2419.96 $[M+Na]^+$, 2434.96 $[M+K]^+$ (calculated for $C_{100}H_{169}BF_2N_{41}O_{25}$: 2396.50)

2.4. BodiFluor-O_2Oc-Tat(48-60)-O_2Oc-Asp(COOH)-resin (**4**)

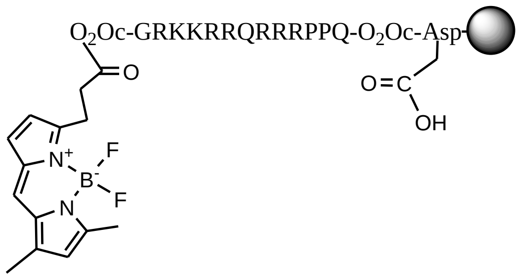

Peptide-resin **2** was treated with 1% (v/v) TFA in CH_2Cl_2 (4 x 10 min) to orthogonally remove the O-2-PhiPr group, followed by neutralization with 5% DIEA in CH_2Cl_2 (4 x 5 min), to give **4**. A mini cleavage would have been inconclusive and was thus omitted.

2.5. BodiFluor-O_2Oc-Tat(48-60)-O_2Oc-Asp(6,3',4',6',2'',5'',3''',4'''-octa-O-acetyl-paromomycin)-resin (**5**)

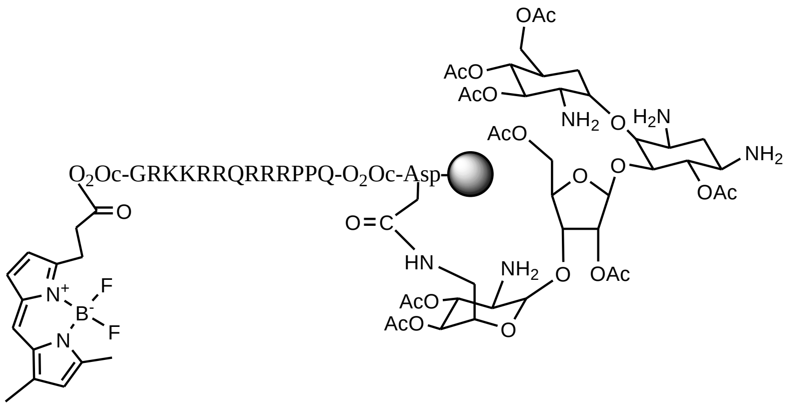

Hydroxyl-protected paromomycin **10** (190.4 mg, 200 µmol) was coupled to the Asp β-carboxyl group of **4** in the presence of HBTU (75.8 mg, 200 µmol) and DIEA (69.68 µl, 400 µmol) in DMF for 2 h, followed by extensive DMF and CH_2Cl_2 washes. A mini cleavage was performed to verify the purity and identity of the resin-bound product.

HPLC t_R 7.17 min (5-60% linear gradient of B into A over 15 min). **ESI-MS** m/z: 821.85 $[M+4H]^{4+}$, 657.75 $[M+5H]^{+5}$, 548.30 $[M+6H]^{+6}$, 470.05 $[M+7H]^{+7}$ (MW calculated: 3278.65).

2.6. BodiFluor-O_2Oc-Tat(48-60)-O_2Oc-Asp(paromomycin)-amide (**6**)

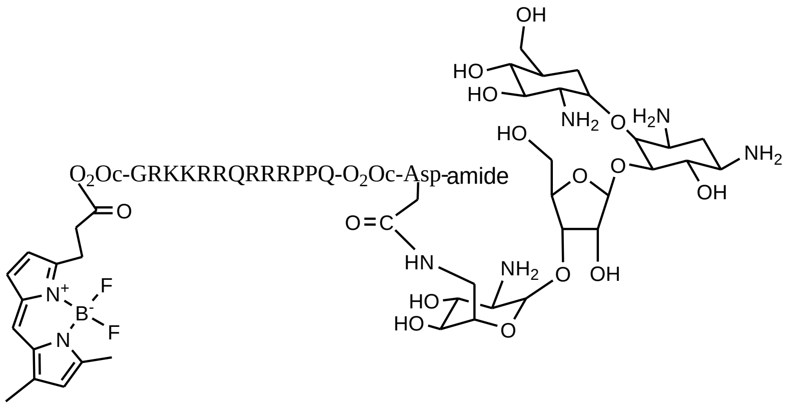

Resin **5** was suspended in 20 mL CH_2Cl_2 and treated with 2 mL of 0.5 M sodium methoxide in MeOH for 2 h, followed by washes with a 0.05 M solution of 15-crown-5 in THF containing 5% (v/v) HOAc to remove sodium ions. The resin was then treated with TFA/H_2O/TIS (95:2.5:2.5 v/v, 90 min) for complete deprotection and cleavage from the resin. The target product was isolated from the cleavage solution by precipitation with chilled diethyl ether, centrifuged for 3 × 10 min at 4ºC, taken up in water and lyophilized. HPLC analysis revealed a complex mixture (Section 3.2.b, black trace) in which only a product with the expected mass of **6** was detected and purified (section 3.2.b, blue trace).

HPLC (SI section 3.2.b.) t_R 6.4 min (5-60% linear gradient of B into A over 15 min). **ESI-MS** (SI section 3.2.b., inset) m/z: 983.40 $[M+3H]^{+3}$, 737.75 $[M+4H]^{+4}$, 590.40 $[M+5H]^{+5}$, 492.20 $[M+6H]^{+6}$, 422.00 $[M+7H]^{+7}$, 369.35 $[M+8H]^{+8}$ (MW calculated: 2944.33). **MALDI-TOF MS** (THAP, positive mode) m/z: 3017.6 $[M+Na]^+$, 3033.6 $[M+K]^+$ (calculated for $C_{123}H_{213}BF_2N_{46}O_{38}$: 2995.13).

2.7. Synthesis of 1,3,2’,2’’’,6’’’-pentakis(N-tert-butyloxycarbonyl)-paromomycin (**8**)

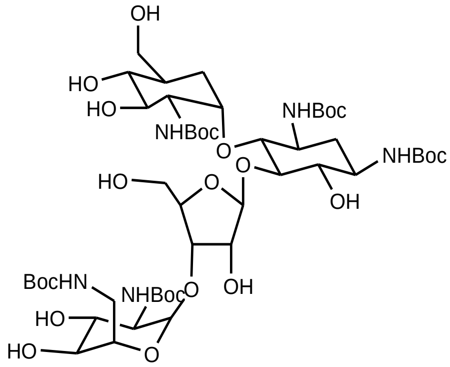

700 mg of paromomycin sulfate **7** (0.981 mmol, 1 eq.) were dissolved in 9.8 mL of water, 4.9 mL of MeOH and 1.642 mL of TEA (11.77 mmol, 12 eq.). Then 3.38 g of Di-*tert*-butyl dicarbonate (14.71 mmol, 15 eq.) dissolved in 4.9 mL of MeOH was added to the stirred

solution by dropwise and the mixture was heated to reflux at 60 °C for 24 h. After cooling, triethylamine and methanol were removed by evaporation. The aqueous layer was extracted with AcOEt (3X10 mL) and the combined organic layers were washed with brine (3X5 mL), dried ($MgSO_4$), filtered and evaporated, rendering a white solid (715.40 mg, 64.13%). That was purified by column chromatography (DCM/MeOH 100:0 to DCM/MeOH 80:20) giving **8** (593.78 mg, 83% yield).

TLC (CH_2Cl_2/CH_3OH, 9:1) Rf 0.5. **^{1}H-NMR** (SI section 3.1.a., DMSO-d_6, 500 MHz, ppm) only most relevant signals: δ = 6.78 (d, J = 5.0 Hz, 1 H), 6.74 (t, J = 10.0 Hz, 1H,), 6.62 (s, 1 H), 6.20 (d, J = 10.0 Hz, 1 H), 5.81 (d, J = 10.0 Hz, 1 H) (5 x NHBoc), 1.35 (s, 45 H, CH_3 *t*Bu). **MALDI-TOF MS** (THAP, positive mode) m/z: 1138.2 $[M+Na]^+$, 1154.2 $[M+K]^+$ (calculated for $C_{48}H_{85}N_5O_{24}$: 1115.56).

2.8. Synthesis of 6,3',4',6',2'',5'',3''',4'''-octa-O-acetyl-1,3,2',2''',6'''-pentakis(N-*tert*butyloxycarbonyl)-paromomycin (**9**)

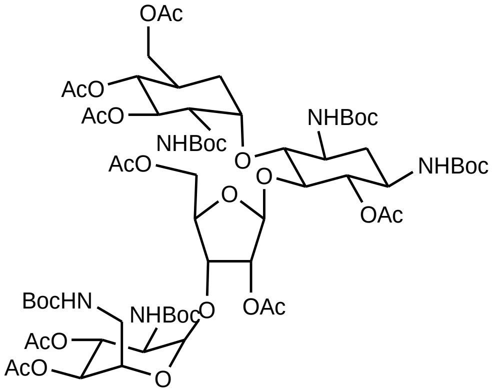

779.85 µL of acetic anhydride (8.25 mmol, 2 x 8OH=16 eq.) were added to a stirred solution containing 575 mg of **8** (0.515 mmol, 1 eq.) dissolved in 2.9 mL of anhydrous pyridine (Aldrich) in the presence of a catalytic amount of *N,N*-dimethylaminopyridine (DMAP). The resultant reaction mixture was stirred at room temperature. After 20 h, it was diluted with 20 mL of DCM and the organic layer was washed with citric acid solution 10% (1x20 mL) $NaHCO_3$ (sat) solution (3x15 mL), brine (3x15 mL), dried (Mg_2SO_4), filtered and concentrated in vacuo. The removal of the solvent rendered a white residue (587.10 mg, 78.44%) that was purified by column chromatography (Hexane/Ethyl acetate 1:1) giving **9** (380 mg, 65% yield).

TLC (hexane/ethyl acetate 1:1) R_f 0.45. **^{1}H-NMR** (SI section 3.1.b., DMSO-d_6, 500 MHz, ppm) only most relevant signals: δ = 6.87 (d, J = 10.0 Hz, 1 H), 6.80 (d, J = 10.0 Hz, 1 H), 6.06 (d, J = 10.0 Hz, 1H,), 5.57 (s, 1 H), 5.38 (d, J = 10.0 Hz, 1 H) (5 x NHBoc), 2.07, 2.04, 1.98, 1.92, 1.86 (5 s, 24H, CH_3O-), 1.35 (s, 45 H, CH_3 *t*Bu). **MALDI-TOF MS** (THAP, positive mode) m/z: 1474.6 $[M+Na]^+$, 1490.6 $[M+K]^+$ (calculated for $C_{64}H_{101}N_5O_{32}$: 1452.51).

2.9. Synthesis of 6,3',4',6',2'',5'',3''',4'''-octa-O-acetyl-paromomycin (**10**)

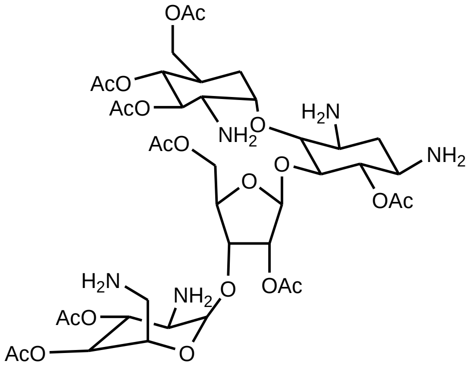

360 mg of **9** (0.248 mmol, 1 eq.) was treated with 36 mL of 40% TFA in CH_2Cl_2 for 2 h at room temperature. Then, CH_2Cl_2 was evaporated and a solid was precipitated by the addition of chilled diethyl ether to remove TFA, centrifuged, taken up in water and lyophilized, giving 236 mg of **10** (100% yield, 25% from paromomycin). After satisfactory characterization by analytical HPLC and MS, it was used without further purification.

HPLC (SI section 3.2.a.) t_R 7.68 min (0-50% linear gradient of B into A over 15 min, 50ºC). **^{1}H-NMR** (SI section 3.1.c., DMSO-d_6, 500 MHz, ppm) only most relevant signals: δ = 2.09, 2.08, 2.07, 1.98, 1.83 (5 s, 24H, CH_3O-), 8.46 (5 x NH_2, broad band). **ESI-MS** m/z: 952.35 $[M+H]^{+1}$, 477.10 $[M+2H]^{+2}$ (MW calculated: 951.92). **MALDI-TOF MS** (THAP, positive mode) m/z: 974.5 $[M+Na]^+$, 990.5 $[M+K]^+$ (calculated for $C_{39}H_{61}N_5O_{22}$: 951.92).

3. Analytical data

3.1. ^{1}H NMR spectra

(a) 1,3,2’,2’’’,6’’’-pentakis(N-tert-butoxycarbonyl)-paromomycin (**8**)

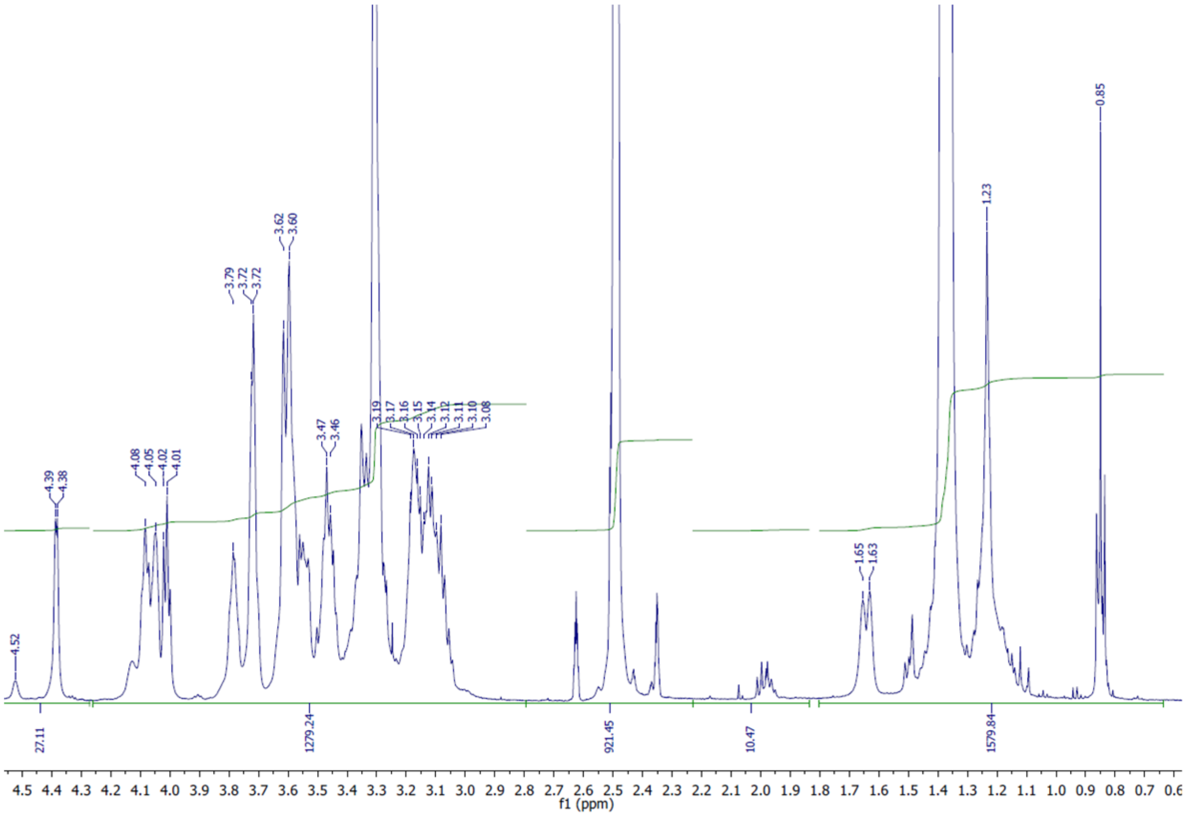

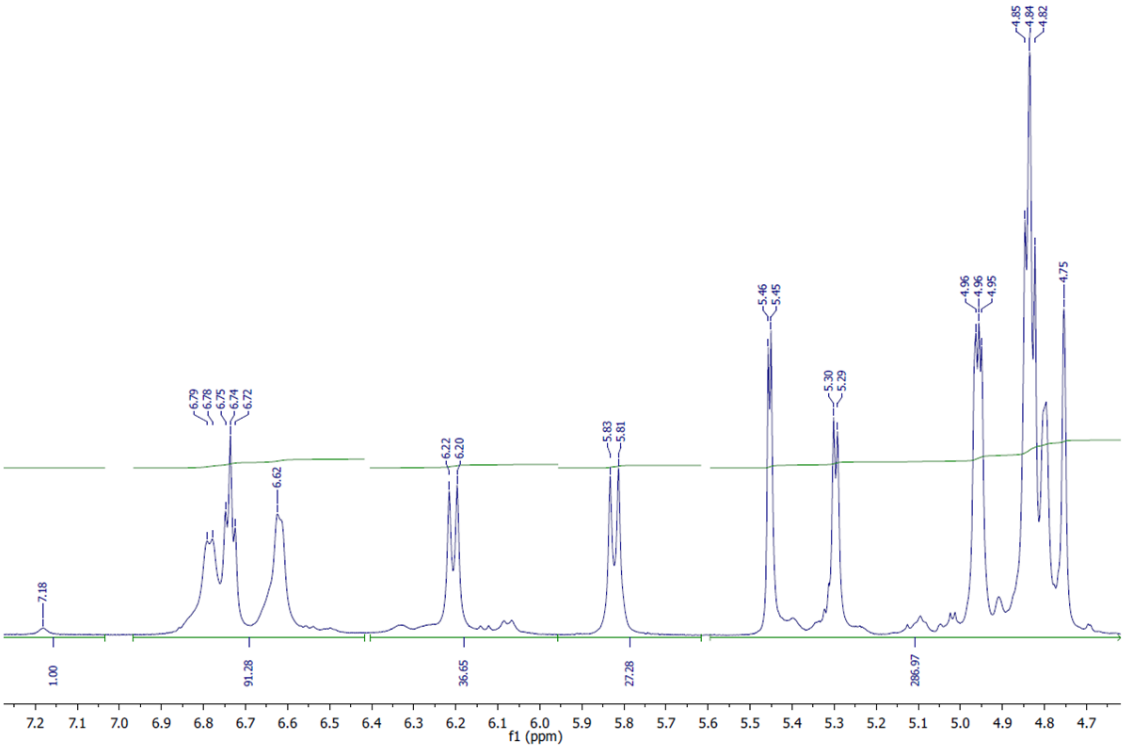

(b) 6,3',4',6',2'',5'',3''',4'''-octa-O-acetyl-1,3,2',2''',6'''-pentakis(N-tertbutoxycarbonyl)-paromomycin (**9**)

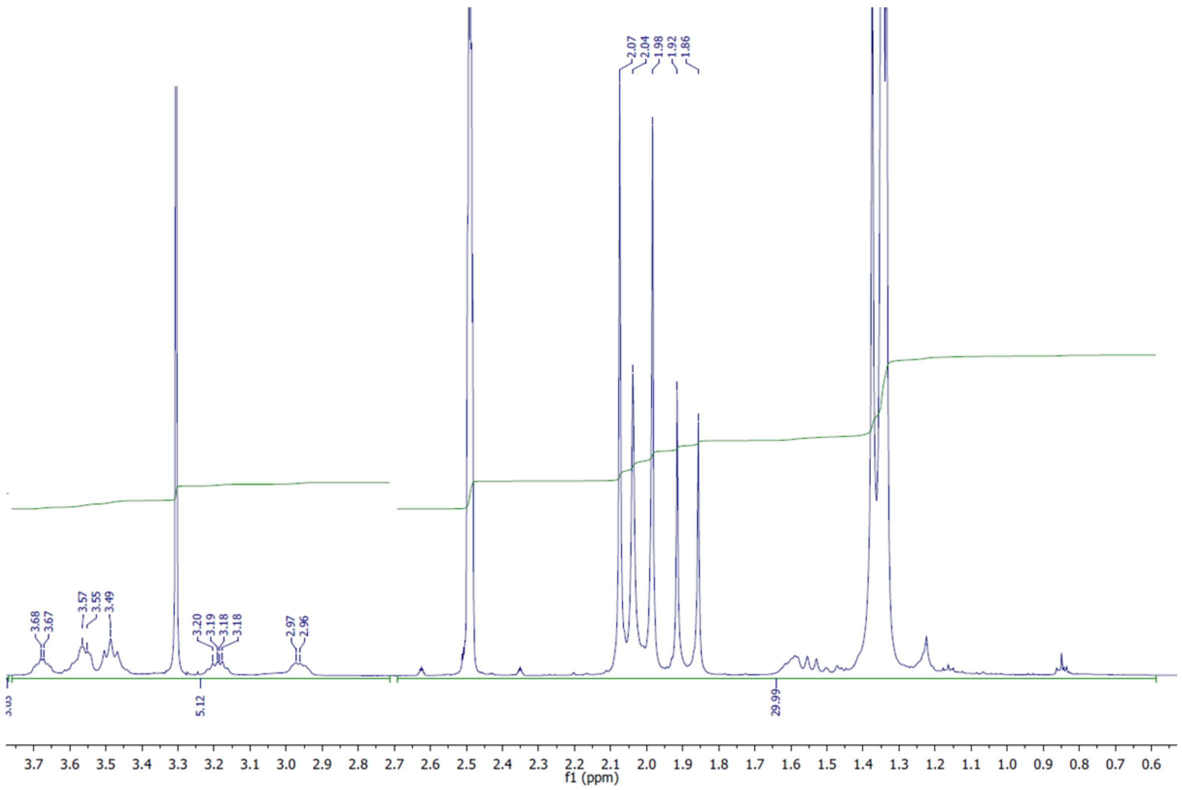

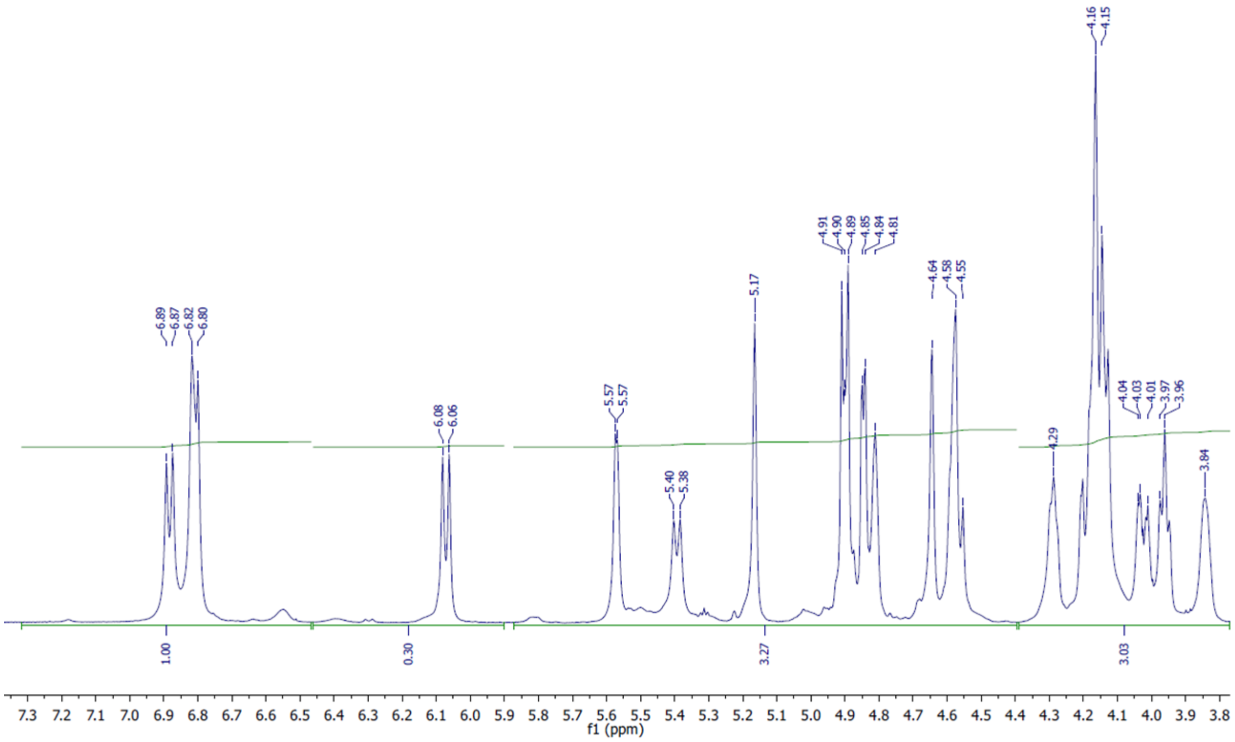

(c) 6,3’,4’,6’,2’’,5’’,3’’’,4’’’-octa-O-acetyl-paromomycin (**10**)

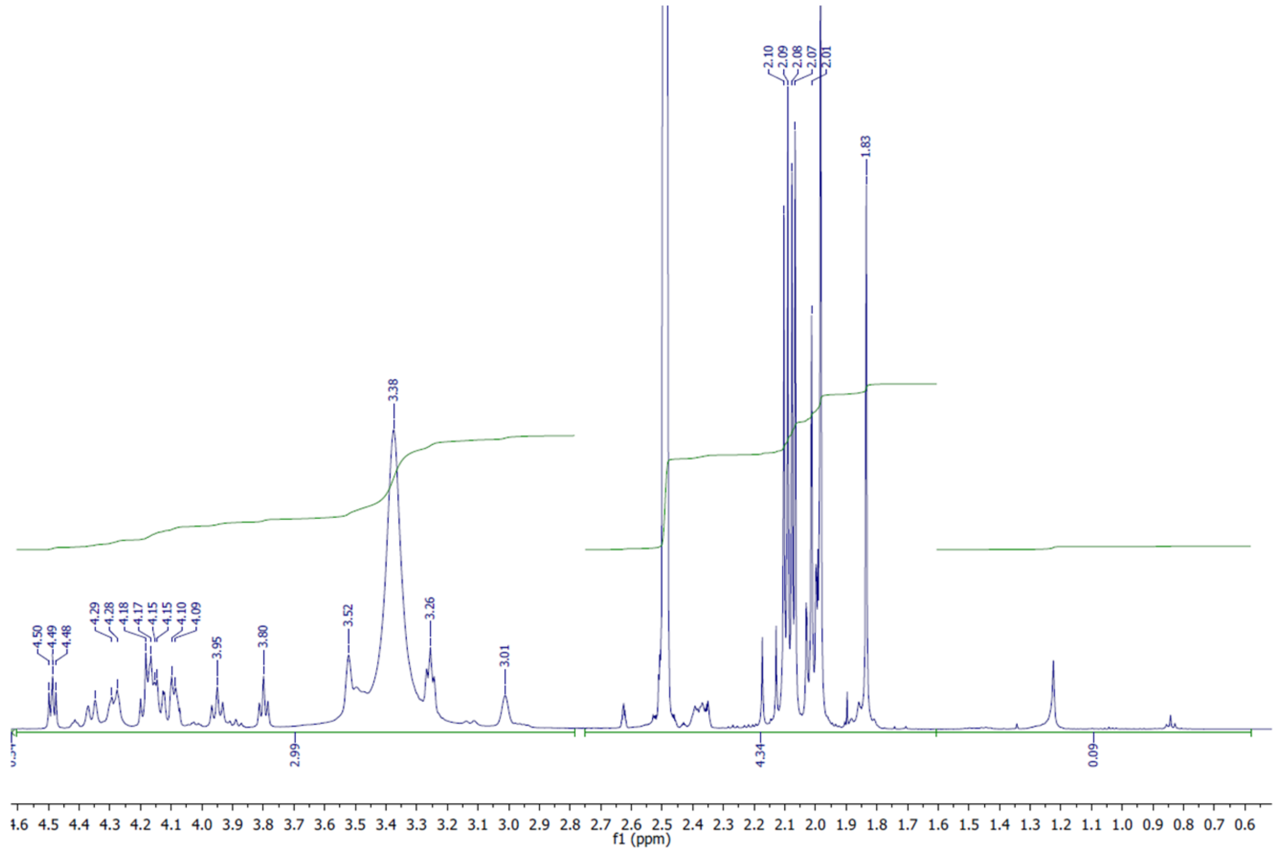

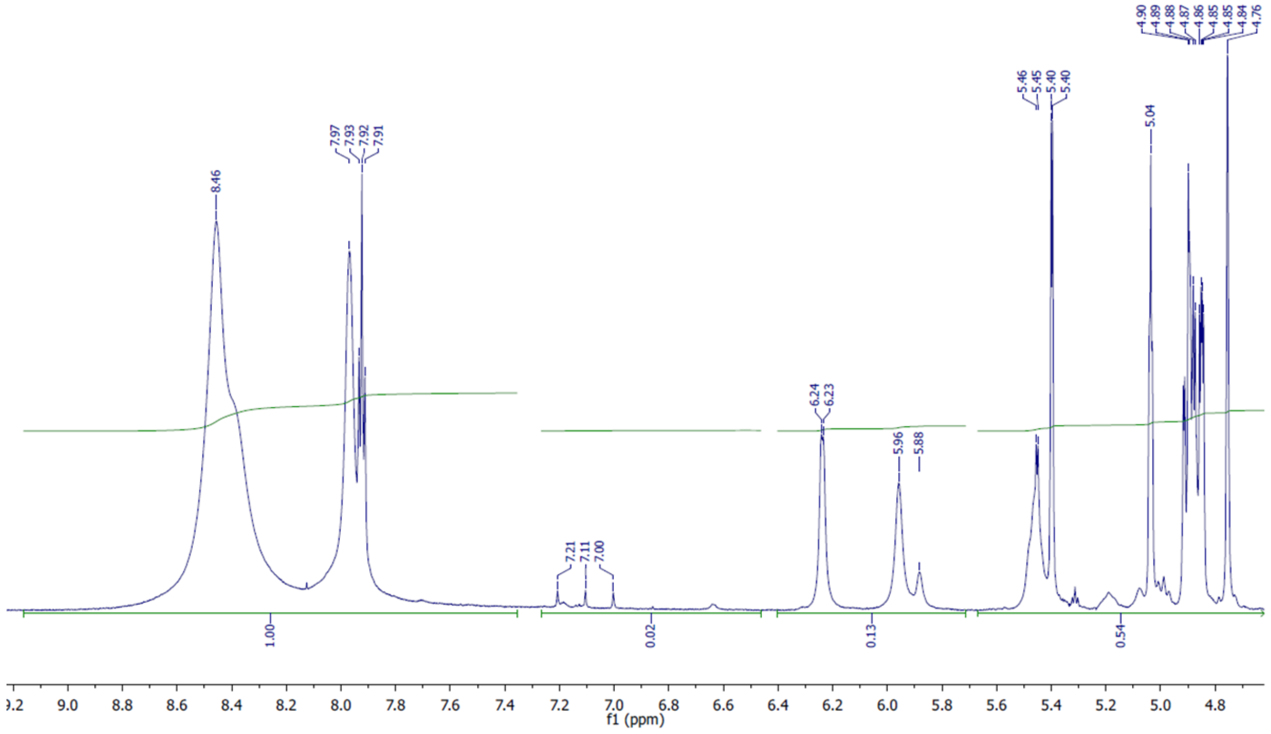

3.2. HPLC chromatograms

(a) 6,3',4',6',2'',5'',3''',4'''-octa-O-acetyl-paromomycin (**10**)

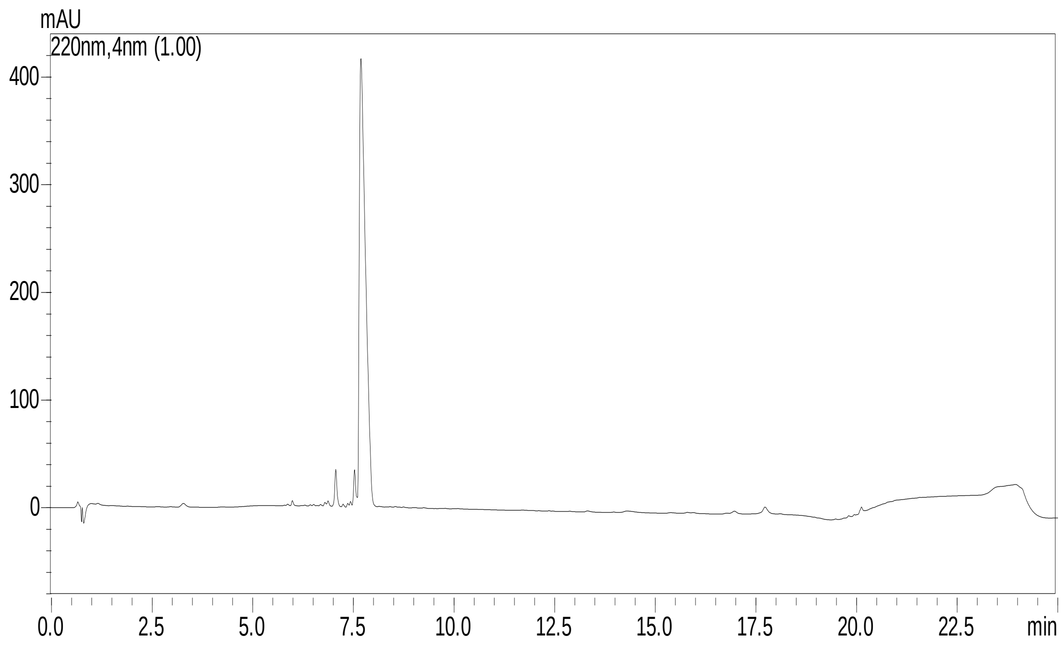

(b) Bodifluor-O_2Oc-Tat(48-60)-O_2Oc-Asp(paromomycin)-amide (**6**)

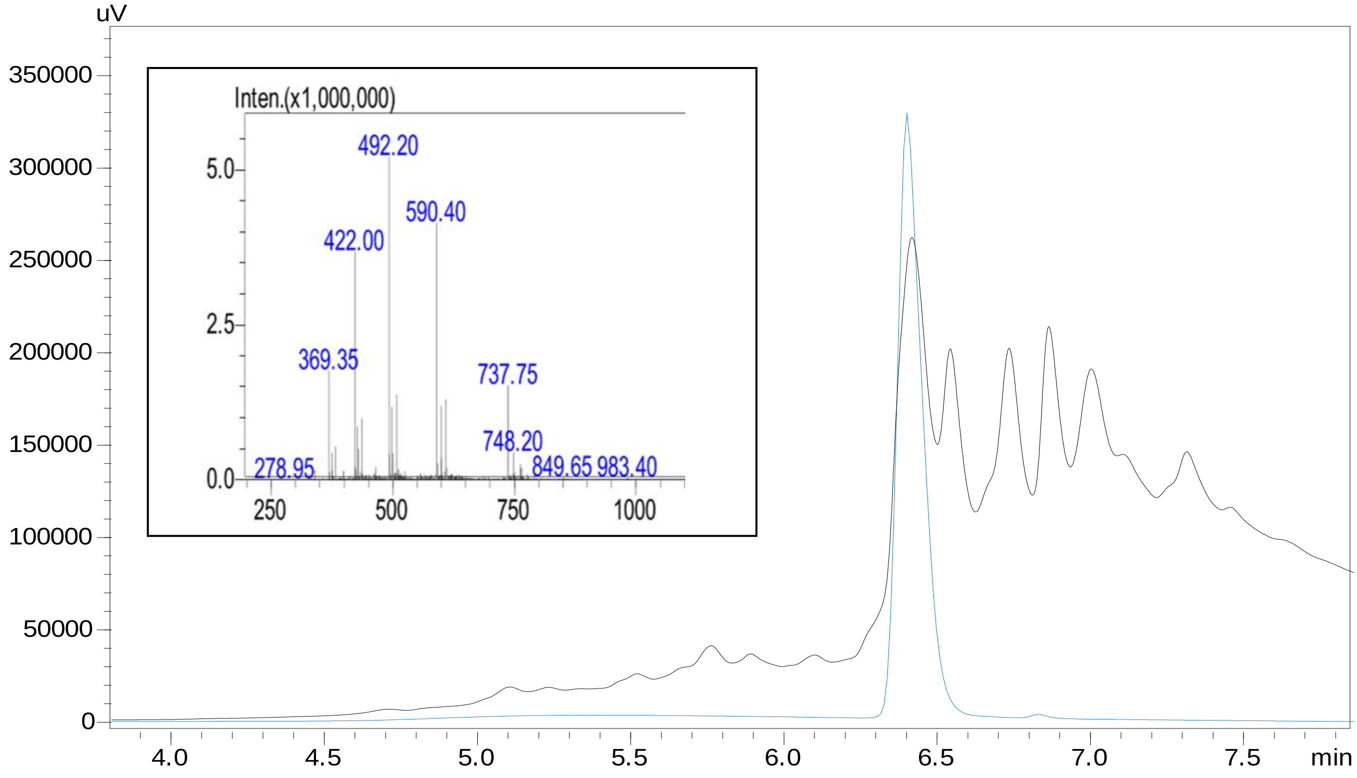

4. Parasite culture and confocal microscopy

Leishmania donovani promastigotes (strain MHOM/SD/00/1S-2D) were resuspended in Hanks' medium supplemented with 10 mM D-glucose (HBSS-Glc) at 2×10^7 cells/mL. Afterwards, 9 µM BodiFluor™ 488-labeled PMM-Tat conjugate was added to the parasite suspension and incubated for 4 h in HBSS-Glc. After incubation, non-incorporated conjugate was removed by washing the cells with HBSS-Glc. Finally, cells were labeled with DAPI (5 µg/mL, 20 min) before their observation, unfixed, in a Leica TCS– SP2 ABOS confocal laser scanning microscope.
